# Medical relevance of UK-funded non-human primate research published from January 1997 to July 2012

**DOI:** 10.1177/0141076814530686

**Published:** 2014-07

**Authors:** Edward Moore

**Affiliations:** Claremount, 9 Porterfield Road, Inverness IV2 3HW, UK

**Keywords:** non-human, primate, research, published, medical, relevance

## Abstract

In 2012, the Bateson Review of research using non-human primates (NHPs) recommended the commissioning of a working group to identify and follow-up the results of UK-funded NHP research of potential benefit for human health (Recommendation 4), but the Medical Research Council (MRC) has postponed implementation of the recommendation. Information on results and potential benefits of NHP research therefore remains unavailable. To fill this gap in knowledge, this study identified all published NHP research studies funded by the MRC, Wellcome Trust and Biotechnology and Biological Sciences Research Council (BBSRC) from January 1997 to July 2012 and assessed full texts for medical relevance. In total, 284 papers were identified, of which 51 (18%) involved invasive NHP research, compared to 176 (61%) which used NHP tissue and cell lines, indicating a shift in research emphasis from invasive whole animal to cell-based research. Of these studies, 98 (35%) were medically relevant, of which 22 had potential therapeutic or public health applications. The relatively low proportion of medical studies together with the small number of applied studies raises questions over the level of investment in medical research and the effectiveness of knowledge transfer from basic to applied research. Implementation of the Bateson Review’s Recommendation 4 would address these questions.

## Introduction

In 2006, a working party led by Professor Sir David Weatherall recommended that UK funders of NHP research undertake a 10-year review of their sponsored research.^1^ In response, the Medical Research Council (MRC), Wellcome Trust and Biotechnology and Biological Sciences Research Council (BBSRC) commissioned a review of all their funded NHP research studies from January 1997 to December 2006. The Review Panel, led by Professor Sir Patrick Bateson, recommended, inter alia, that ‘Science policy-makers together with the public sector, private sector and charitable funders of research should commission a working group to develop proposals for a mechanism (output-scanning) to identify research results with potential to deliver improvements to healthcare or other significant benefits to society, and to assess the extent to which the potential benefits are achieved’ (Recommendation 4).^2^ The MRC has indicated that this recommendation ‘is not currently being taken forward, as we have prioritised work on other recommendations in the Bateson Review’. Consequently, factual information on NHP research results and their medical relevance remains unavailable to the public and wider medical profession. This study provides this information.

## Methods

The Royal Society of Medicine (RSM) Library search services team was commissioned to undertake a literature search encompassing all NHP research sponsored by the MRC, Wellcome Trust and BBSRC published in UK scientific or medical journals indexed by Medline from January 1997 to July 2012.

Access to full texts was sought using Europe PubMed Central, Internet search engines, Athens and the RSM Library e-journal resource in turn, failing which access was purchased through the RSM Library. Full texts were scrutinised for medical relevance.

### Definitions of NHP research categories

- Medical: aimed at a specific human disease or health problem;
- Veterinary: aimed at a specific animal disease or health problem;
- Biological: NHP research excluding medical and veterinary studies.

## Results

### Literature search

This identified 286 study abstracts. One study comparing types of ultrasound equipment and one funded by the Medical Research Council of Australia were excluded. Access to the remaining 284 full texts was gained using Europe PubMed Central (155 studies), Internet search engines (75), Athens (42) and the RSM Library e-journal resource (8). Access to the remaining four was purchased through the RSM Library.

### Research funding

Research funders were identified in study abstracts. Funding of biological, medical and veterinary research by the UK funders, alone and in combination, is shown in Table 1.

**Table 1.** Published NHP research studies funded by the MRC, Wellcome Trust and BBSRC, by research category, January 1997 to July 2012.

Funder	Research category			
	Biological	Medical	Veterinary	All
Wellcome Trust	91	63	7	161
MRC	25	16	2	43
BBSRC	37	2	3	42
Wellcome Trust + MRC	9	8	1	18
Wellcome Trust + BBSRC	4	2	0	6
MRC + BBSRC	3	1	0	4
Wellcome Trust + MRC + BBSRC	4	6	0	10
All	173	98	13	284

Forty-six studies were also funded by the US National Institutes of Health (30 studies, of which three were co-funded by Howard Hughes Medical Institute), Cancer Research UK (8), British Heart Foundation (3), UK Department of Health (2) or Telethon of Italy (3). In 95 studies, researchers acknowledged additional support from grants, fellowships, studentships, gifts of research materials from fellow scientists, and sponsorship from charities, universities, the pharmaceutical industry and national and local governments in the UK, Europe, the USA, Africa, China and Australia. Of 55 studies based outside the UK, 27 were in Europe, 18 in the USA, three in China, three in Africa, two in Australia and one each in Thailand and Japan.

### Types of research

Research was grouped into invasive and non-invasive whole animal and cellular research. Invasive whole animal studies involved either marmosets or macaque monkeys and, in one study, baboons. Non-invasive research also concerned gorillas, bonobos, chimpanzees and gibbons. Cellular research utilised NHP tissue and COS-1, COS-7 and Vero cell lines derived from the African green monkey (*Cercopithecus aethiops*); 40 of these studies also involved invasive research in rodents, cattle, goats, sheep, poultry or fish (Table 2).

**Table 2.** NHP whole animal and cellular studies by research category and invasiveness.

Research category	Whole animal studies		Cellular studies	
	Invasive	Non- invasive	Invasive	Non- invasive
Biological	43	50	16	64
Medical	5	7	19	67
Veterinary	3	0	5	5

### Biological studies

Invasive whole animal studies focused on neurobiology (22 studies), reproduction (10), vision (9) and pharmacology (2). Non-invasive studies concerned neurobiology (19 studies), evolutionary biology (12), behaviour (5), genomics (5), vision (5), physiology (3) and reproduction (1).

Cellular studies focused, *inter alia*, on protein metabolism (46 studies), gene expression and function (36), endoplasmic reticulum function (6), phosphorylation (6), signal transduction (6), calcium metabolism (5), microtubule metabolism (5), the Golgi apparatus (5), calcium channel function (4), cell membrane function (4), phagocytosis (4), receptor trafficking (3), cell movement (2), cell adhesion (2), stress granule formation (1) and oxidative stress (1).

### Supplemental file 1

This provides a chronological list of references to the 80 cellular studies.

### Veterinary studies

Invasive whole animal studies focused on infection by *Plasmodium knowlesi* (1 study), simian immune deficiency virus (1) and an outbreak of *Mycobacterium intracellulare* in a captive colony of wild baboons (1).

Cellular research focused on disease pathogenesis (5 studies), control of infection (2) and vaccine development (3).

### Medical studies

Invasive whole animal studies focused on chronic obstructive pulmonary disease (2 studies), measles (1), gene transfer (1) and testing a neurosurgical procedure (1). Non-invasive studies concerned HIV infection, HIV vaccines, hepatitis C, animal models of trypanosomiasis, evolution of malaria, obesity and gender-related behaviour (1 each). Cellular studies focused mainly on genetic disorders, infectious disease and cancer. Table 3 presents the 98 medical studies by research subject and area of medical relevance.

**Table 3.** NHP medical research publications by research subject and area of medical relevance, January 1997 to July 2012.

Research subject	Medical relevance		
	Pathogenesis	Potential therapy	Public health
Genetic disorders	31	6	0
Virology	19	4	1
Bacteriology	7	1	2
Parasitology	6	3	1
Cancer	11	2	0
Bronchodilator therapy	0	1	0
Testing neurosurgical technique	0	1	0
Gender-related behaviour	1	0	0
Obesity	1	0	0

### Genetic disorders

#### Pathogenesis research

This focused on alpha 1-antitrypsin deficiency, Alzheimer’s disease, Bardet-Biedl syndrome, cardiovascular disease, chronic obstructive pulmonary disease, craniosynostosis, cystic fibrosis, diabetes Type 2, familial encephalopathy with neuroserpin inclusion bodies (FENIB), frontotemporal dementia with Parkinsonism linked to chromosome 17 (FTDP-17), Frasier syndrome, fragile X-associated tremor ataxia syndrome (FXTAS), hereditary sensory and autonomic neuropathy Type 5 (HSAN5), Huntington’s disease (HD), myoclonus dystonia syndrome, oculopharyngeal muscular dystrophy, Parkinson’s disease, popliteal pterygium syndrome, retinitis pigmentosa, retinoschisis, schizophrenia, Treacher Collins syndrome, Van der Woude syndrome, Williams-Beuren syndrome and Wolfram syndrome.

#### Studies of therapeutic relevance

- Davies *et al.*^3^ investigated the effect of trehalose in cell and transgenic mouse models of the late onset autosomal disease oculopharyngeal muscular dystrophy. The drug reduced aggregate formation and toxicity of mutant protein in cell models and oral administration attenuated muscle weakness in mice: ‘As one can identify almost all cases at risk, this disease is amenable to pre-symptomatic treatment … Trehalose would be particularly attractive for this strategy, given its safety and suitability for long term use.’
- Sarkar *et al.*^4^ demonstrated that combined treatment with lithium and rapamycin in a drosophila fly model of HD enhanced macroautophagy of aggregate-prone proteins, providing proof-of-principle of this combination as a potential treatment for HD.
- Ravikumar *et al.*^5^ showed that inhibition of the endosomal protein Rab5 enhanced polyglutamine toxicity in cell and fly models of HD, whereas overexpression attenuated toxicity: ‘A possible therapeutic approach for HD and other proteinopathies would thus be to enhance Rab5 activity’.
- Vamathevan *et al.*^6^ investigated the role of positive gene selection in the higher incidence in humans of diseases including epithelial cancer, Alzheimer’s disease and auto-immune diseases: ‘Understanding the evolutionary history of disease genes can also significantly impact the choice of pre-clinical animal models in the drug discovery process’.
- Wang *et al.*,^7^ in an international collaborative study, presented the first diploid genome sequence of an Asian individual (YH). They identified several genotypes which conferred an increased risk for YH of tobacco addiction and Alzheimer’s disease. They considered that ‘personal genome sequencing may eventually become an essential tool for diagnosis, prevention and therapy of human diseases’.
- Underwood *et al.*^8^ found that in fly and zebrafish models of HD, antioxidants ‘exacerbate the disease phenotype and abrogate the rescue seen with autophagy-inducing agents. Thus, the potential benefits in neurodegenerative diseases of some classes of antioxidants may be compromised by their autophagy-blocking properties.’

### Virology

#### Pathogenesis research

This focused on Bunyamwera virus, human cytomegalovirus major, severe acute respiratory syndrome coronavirus, enterovirus 71, hepatitis C virus, herpes simplex virus type 1, herpesvirus-6, HIV-1 Group P, measles virus, mumps vaccines, mumps virus, parainfluenza virus type 5, retroviral restriction, Rift Valley fever virus, Semliki Forest virus and vaccinia virus.

#### Studies of therapeutic or public health relevance

- Howard *et al.*^9^ investigated the use of herpes simplex virus, lacking functional genes for ICP 27 and ICP34.5, as a vector to deliver a marker gene to rodent and NHP central nervous systems. This produced relatively minimal damage, so offering ‘a platform for the development of vectors which are sufficiently safe for ultimate use in human gene therapy’.
- Griffiths *et al.*^10^ investigated the requirements for herpesvirus saimiri (HVS) episomal persistence: ‘This analysis will help towards the development of safe replication-disabled HVS-based vectors for gene therapy applications, such as the HVS amplicon system’.
- Roberts and Jopling^11^ reviewed the treatment of chimpanzees suffering from chronic hepatitis C (HCV) with the micro-RNA inhibitor SPC3649. This reduced viral load and improved liver histology, ‘suggesting that damage induced by HCV infection might be reparable’. They proposed that such an approach ‘might have therapeutic potential in humans’.
- Kelly *et al.*^12^ designed a series of peptides and tested them for antiviral and antibacterial activity: ‘Our finding that apoBdpL/R-W had broad activity against herpes viruses and different HIV strains yet minimal cytotoxicity or haemolytic activity, supports its use as a lead for the development of microbiocides or HIV therapeutics’.
- Fast and Kaleebu^13^ reviewed international progress towards developing an effective AIDS vaccine: ‘An important need, in the complex environment of today, is for open communication and collaboration between different institutions, vaccine developers, and investigators who are conducting or wish to conduct vaccine trials, in particular efficacy trials’.

### Bacteriology

#### Pathogenesis research

This focused on *Burkholderia pseudomallei*, *Clostridium difficile*, *Escherichia coli*, *Helicobacter pylori*, *Salmonella Dublin*, *Mycobacterium tuberculosis* and rickettsial phylogeny.

#### Studies of therapeutic or public health relevance

- Lamden *et al.*^14^ investigated the effect of penicillin on *Chlamydia trachomatis* DNA replication and found that the antibiotic blocked binary fission but ‘did not prevent chromosomal or plasmid DNA replication’.
- Stabler *et al.*^15^ investigated the worldwide rise in *Clostridium difficile* infection and its increasing virulence: ‘These studies facilitate pinpointing the genetic and phenotypic attributes that may explain the emergence of the hypervirulent 027 strain and contribute in general to our understanding of the evolution of bacterial virulence’.
- Salisbury *et al.*^16^ found that Salmonella Virchow infection of chickens can occur without overt clinical signs: ‘The asymptomatic colonization of chickens indicates an increased ability of S. Virchow to enter the food chain undetected’.

### Parasitology

#### Pathogenesis research

This focused on malaria, trypanosomiasis and toxoplasmosis.

#### Studies of therapeutic or public health relevance

- Biagini *et al.*^17^ considered how newfound knowledge of parasite transport physiology could be turned into a strategy for drug delivery in malaria.
- Kennedy^18^ argued in favour of the continued use of animal models of human African trypanosomiasis, despite their limitations.
- Wilson and Coulson^19^ reviewed the strategies used by the schistosome in combating the immune response: ‘the animal models suggest that targeting multiple functions is more likely to achieve worm elimination, thereby requiring administration of a cocktail of antigens’. They concluded: ‘On a personal note, after nearly 30 years research on schistosome vaccines, we still hope that a magic bullet akin to the Taenia onchosphere antigens will be found by serendipity. Experience tells us that more likely, the laudable goal will require a “long march”.’
- Liu *et al.*^20^ investigated the origin of human *Plasmodium falciparum* malaria by identifying and characterizing Plasmodium spp. DNA sequences in faecal samples from wild-living apes: ‘Importantly, all currently available human *P. falciparum* sequences comprised a single lineage nested within the G1 clade of gorilla parasites. This finding indicates that human *P. falciparum* is of gorilla origin and not of chimpanzee, bonobo or ancient human origin.’

### Cancer

#### Pathogenesis research

This focused on cancers of the breast, prostate and ovary; leukaemia; neuroblastoma; tumour suppression; hypoxia regulatory factors and chromosomal changes.

#### Studies of therapeutic relevance

- Chu *et al.*,^21^ in their study of the effects on apoptosis of phosphatidylinositol 4-kinases, concluded that, for certain breast cancers: ‘targeted inhibition of PI4KIIIβ represents a potential chemotherapeutic strategy to treat such malignancies’.
- Dart *et al.*^22^ investigated the relationship of the corepressor protein prohibitin and the androgen receptor (AR) in prostate cancer and concluded that ‘altering AR activity via increasing levels or activity of corepressors is a valid therapeutic strategy for advanced prostate cancer’.

### Miscellaneous studies

#### Pathogenesis research

This focused on obesity and gender-related behaviour.

#### Studies of therapeutic relevance

- Buckley *et al.*,^23^ in their investigation of airway relaxation in response to inhaled prostaglandin E_2_, found that in human beings it was the EP_4_ receptor which mediated airway relaxation and proposed that ‘EP_4_ selective compounds could potentially be the next generation of bronchodilators’.
- Pereira *et al.*^24^ reported on their preliminary research and use of deep brain stimulation in patients with Parkinson’s disease in whom symptoms of gait freezing and postural instability predominate.

### Supplemental file 2

This provides a chronological list of references to all 98 medically relevant studies.

## Discussion

The study findings provide insights into three aspects of UK-funded NHP research.

### An apparent shift in emphasis from invasive whole animal to cellular research

Overall, only 51 (18%) of the studies involved invasive research on living NHPs, compared to 176 (61%) which made use of NHP tissue and cell lines. The writer could find no comparable data from previous years, but it is submitted that this represents a change in research emphasis from invasive whole animal studies to cell-based molecular and genetic research. In particular, the change means that basic medical and biological research now share common ground in respect of cellular research methods and materials, offering the opportunity for coordination of biological studies of normal cell function with medical studies of cell function in disease.

The reduction in invasive NHP studies should reduce harm to animals and will hopefully lead to a reduction in the level of opposition to NHP research and aggression towards researchers, as well as encouraging charitable support. The shift in research emphasis is also relevant to the ethical debate: overall, 193 (68%) of the studies were non-invasive in NHPs or other animals and therefore in accord with the Beauchamp and Childress principle of non-maleficence^25^ and the medical axiom of ‘First do no harm’.

### The proportion of research of medical relevance

At 35%, the proportion of medical studies was low compared to biological studies (61%). This, however, is broadly in line with the findings of the Cooksey Review of 2006, which cited the UK Clinical Research Collaboration analysis showing that around two-thirds of public and charitable funding of health research was invested in basic science projects. The Review considered that current funding levels for basic science should be maintained, but recommended: ‘that future increases in funding should be weighted towards translational and applied research until a more balanced portfolio is achieved’.^26^ Both Government and research funders will be aware of the recommendation and investment in medically relevant NHP research will hopefully rise to a level equivalent to that of basic biological research.

### Knowledge transfer from basic medical to applied research

The level of translation from basic to applied research is a touchstone of research effectiveness and provides an assessment of return on research investment. The small number (22 including three reviews) of applied medical studies was therefore a cause for concern. If this is a fair representation of translation of basic research from earlier years, it suggests that research results may not always have been taken forward into applied studies, echoing the Bateson Review Panel’s finding that ‘some evidence was provided of the translation of fundamental science into applied science and practical application, but in many cases too little consideration was given to effective knowledge transfer’.^2^ On the other hand, the low level of translation may have been due, at least in part, to legitimate negative research findings. There is insufficient information on which to draw conclusions. Whatever the reasons, in order to determine the true level of translation, it is important that all studies of potential value for human health are identified and followed up. Implementation of the Bateson Review’s Recommendation 4 would meet this need.

### Future outlook

The shift in research emphasis from invasive whole animal to cellular research brings medical and biological science into a complementary relationship, which will hopefully lead to better understanding of disease aetiology, and improved diagnosis, prevention and treatment, particularly of chronic disease. It also presents an opportunity for researchers and research funders to engage in a more open relationship with the public.

With the growing research opportunities in molecular biology and genetics, the need for coordination of biological and medical research may increase. The need for identification and follow-up of studies of potential importance for human health will remain.

## Conclusions

This paper raises questions over the level of investment in medically relevant NHP research, the effectiveness of knowledge transfer from basic to applied research and the coordination of biological and medical research. Implementation of the Bateson Review’s Recommendation 4 would address these questions.

## Declarations

### Guarantor

EM

### Provenance

Not commissioned; peer-reviewed by Ian Olver
